# What’s the Matter with Riggs?

**Published:** 1877-12

**Authors:** Sam Jones


*To the Editor of the Dental Register:*

Dear Sir:—I am dying—to know what is the matter with Riggs. I thought he was sound; but, from a glance at the journals, I see he has a disease. Did he catch it at Chicago? I hear that it was talked about there, and that is a fearful place. An acquaintance of mine caught a dreadful disease at Chicago, some years ago, and has been afraid to go back again. If Riggs caught his disease there he'll be more timid still.

What's the matter with Riggs, anyway? Tell a fellow, will you? Doctor Sykes, my family physician, thinks it was brought on him by the misconduct and ingratitude of his students, which may be true; for I have seen boys bring their father's gray hairs with sorrow to the grave, and teachers often dearly love their pupils. I had a pupil myself, once. I loved him as a son, and was sad if he sneezed. The little demon proved wayward, and ran off; and now I am, wrinkled and gray, grieving about that boy. Do you think I have Riggs' disease?

When a man is really ill, it is cruel to insinuate that his illness is all pretence. What an outrage, then, to speak of "Riggs' disease, so called." My cousin, John Jones, has a disease—hypochondria, really, but laziness, "so called." At least, every body calls it that. Now, Riggs is not that kind of a man. He has a disease, but what is it?

I infer that Riggs' students regard scraping as good for his disease. Then why do they let their kind preceptor suffer, if scraping will cure him? John Blixon had a disease,—his teeth were loose, and covered with calcareous deposits, his gums were swollen, spongy, tender and irritable, his throat was "an open sepulcher;" but he was cured, scraping being one factor in the treatment. But I never thought of calling his complaint "Blixon's disease," because he had it, nor "Jones' disease," because I cured him. But who cares for Blixon? What's the matter with Riggs? That's what I want to know.

But after all, how much our profession owes to diseased men! If Atkinson's external vision had not been slightly weakened, he would, probably, to-day, be a country preacher, with no higher reputation than Moody and Spurgeon, and his inward sight would not have become strong enough to see through millstones and mountains; and he would have failed to tell us four thousand and three things that we wanted to know. Taft got crippled when he was little, or he would, most likely, have been a gunsmith; then who would have taught us operative dentistry in two colleges? And what would have become of you, Mr. REGISTER? Watt, too lame to limp, gave us the warm air syringe; and Riggs, in spite of his disease, has given us a half dozen or more very valuable instruments. God bless the diseased, say I; but let us not parade their diseases in public meetings.

And now, with respect for Riggs, and with my good wishes for yourself, I am—but, say! what's the matter with Riggs?

SAM. JONES.

